# Cannabisgebrauch bei Jugendlichen: Übersicht und Positionspapier der Arbeitsgruppe „Suchtstörungen im Jugendalter“ der Österreichischen Gesellschaft für Kinder- und Jugendpsychiatrie, Psychosomatik und Psychotherapie (ÖGKJP)

**DOI:** 10.1007/s40211-022-00424-1

**Published:** 2022-07-28

**Authors:** Katrin Skala, Thomas Trabi, Martin Fuchs, Ralf Gössler, Christian-Wilhelm Haas-Stockmair, Nicole Kriechbaumer, Monika Leitner, Nora Ortner, Melanie Reiter, Christian Müller, Wolfgang Wladika

**Affiliations:** 1 Univ. Klinik für Kinder und Jugendpsychiatrie, Medizinische Universität Wien, Währinger Gürtel 18–20, 1090 Wien, Österreich; 2 Abteilung für Kinder- und Jugendpsychiatrie, LKH Graz II, Graz, Österreich; 3 Univ. Klinik für Kinder- und Jugendpsychiatrie, Medizinische Universität Innsbruck, Innsbruck, Österreich; 4 Abteilung für Kinder- und Jugendpsychiatrie, Klinik Floridsdorf, Floridsdorf, Österreich; 5 Univ. Klinik für Kinder- und Jugendpsychiatrie, Kepler Universiätsklinikum, Linz, Österreich; 6 Sucht- und Drogenkoordination Wien, Wien, Österreich; 7 Praxis für Kinder- und Jugendpsychiatrie, Graz, Österreich; 8 Ambulatorium für Kinder- u. Jugendpsychiatrie, PSD Einsenstadt, Einsenstadt, Österreich; 9 Abteilung für Neurologie und Psychiatrie des Kindes- und Jugendalters, Klinikum Klagenfurt, Klagenfurt, Österreich

**Keywords:** Minderjährige, Substanzkonsum, Epidemiologie, Gehirnentwicklung, Behandlung, Minors, Substance use, Epidemiology, Brain development, Therapy

## Abstract

**Fragestellung:**

Cannabis ist die, von österreichischen Jugendlichen am häufigsten konsumierte, illegalisierte Droge. Aufgrund der, in den letzten Jahren erfolgten schrittweise Entkriminalisierung bis hin zur Legalisierung in vielen europäischen Ländern möchte die ÖGKJP auf ausgewogene und wissenschaftlich basierte Art und Weise Stellung zur komplexen Thematik des Cannabisge- und Missbrauchs bei Jugendlichen beziehen.

**Methodik:**

Die Medline mit jeweils dem spezifischen Unterthema angepassten Suchen auf aktuelle Studien durchsucht. Weiters wurden aus anerkannten Kompendien zitiert.

**Ergebnisse:**

Während gelegentlicher Freizeitkonsum von Cannabis bei Erwachsenen mit abgeschlossener Hirnreifung und ohne Risikoprofil für psychische Störungen relativ harmlos sein dürfte, können frühzeitigem Konsumbeginn mit regelmäßigem Konsum sowie die zunehmend verfügbaren, hochpotenten Cannabis-Sorten zu expliziten und zum Teil irreversiblen neurokognitiven Hirnfunktionsstörungen führen.

**Schlussfolgerung:**

Eine gesetzliche Freigabe des Cannabis-Konsums für Jugendliche aufgrund der Risken der zu erwartenden Schäden im Bereich der Gehirnentwicklung abzulehnen. Zugleich gilt es aber, vernünftige gesetzliche Regelungen zu etablieren, die der Tatsache, dass über 30 % aller europäischen Jugendlichen gelegentlich Cannabis konsumieren, adäquat begegnen kann. Wir sprechen uns hier auch klar dafür aus, Cannabiskonsumenten nicht zu kriminalisieren und gefährdeten und suchtkranken Cannabiskonsumenten die benötigte Unterstützung zukommen zu lassen.

## Abstract (en)

**Background:**

Cannabis is the illegal drug most frequently used by Minors in Austria. Due to the gradual decriminalization and legalization that has taken place in many European countries in recent years, the ÖGKJP would like to take a balanced and scientifically based stand on the complex issue of cannabis use and abuse among young people.

**Methods:**

The authors searched the medline for current studies using searches tailored to each specific subtopic. Furthermore, recognized compendiums were quoted.

**Results:**

While occasional recreational use of cannabis in adults with completed brain maturation and no risk profile for mental disorders is likely to be relatively harmless, early initiation of use with regular use and the increasingly available, highly potent cannabis varieties can lead to explicit and sometimes irreversible neurocognitive brain dysfunction.

**Conclusion:**

Legalisation of cannabis consumption for minors needs to be objected to due to the risks of the expected damage in the area of brain development. At the same time, however, it is important to establish sensible legal regulations in order to be able to adequately counteract the fact that over 30% of all European young people occasionally consume cannabis. We are also clearly recommending to not criminalize cannabis users and provide necessary support to vulnerable and addicted cannabis users.

## Einleitung

Cannabis ist die, von österreichischen Jugendlichen nach wie vor am häufigsten konsumierte illegalisierte Droge. In den letzten Jahren erfolgte in vielen europäischen Ländern, aber auch in einigen US-amerikanischen Bundesstaaten, eine schrittweise Entkriminalisierung bis hin zu einer Legalisierung des Cannabis-Gebrauchs bei Erwachsenen. Dies soll den therapeutischen oder medizinischen Gebrauch von Cannabis-Produkten ermöglichen, zum Beispiel bei der Bekämpfung von therapieresistenten Schmerzen. In vielen Gebieten wurde aber auch der so genannte „recreational use“ (Freizeitnutzung) von Cannabis erlaubt. Dadurch ist im Hintergrund eine potente Cannabis-Industrie entstanden, die sich unter anderem für die Entwicklung von Cannabis-Sorten mit hohem THC-Gehalt oder für die Entwicklung von Cannabis-Extrakten mit sehr starker Wirkung verantwortlich zeigte. All diese Angebote richten sich bisher an Erwachsene, aber es ist natürlich davon auszugehen, dass auch junge Menschen diese veränderten Konsumformen nutzen.

Vor diesem Hintergrund möchte das Positionspapier der ÖGKJP auf ausgewogene und wissenschaftlich basierte Art und Weise Stellung zur komplexen Thematik des Cannabisge- und Missbrauchs bei Jugendlichen beziehen und die vorhandenen Informationen und Daten kompakt zusammenfassen, um damit eine fundierte Basis für weitere Diskussionen zu schaffen.

Dieses Positionspapier soll auch ein Aufruf sein, Vertreter^1^ der Kinder- und Jugendpsychiatrie in politische Entscheidungen einzubinden, um sinnvolle und praktikable Ansätze zur Prävention und Versorgung substanzkonsumgefährdeter Kinder und Jugendlicher mitzugestalten.

## Geschichte, Wirkungen, Züchtungen, Konzentrationen, Marktlage & Nachweisbarkeit

### Zur Geschichte von Cannabis

*Cannabis sativa* ist eine der ältesten Kulturpflanzen [1]. Bereits ca. 2737 v. Chr. wird Hanf als Heilmittel erwähnt. Es wird u. a. gegen Gicht, Rheuma und Verstopfung empfohlen. Es gibt Hinweise auf die Nutzung von Hanf in Europa und Asien ab dem frühen Holozän (ca. 8000 v. Chr.) [2].

Im 19. Jahrhundert, als Cannabis im Rahmen der Kolonialisierung Afrikas und Asiens vermehrt seinen Weg nach Europa fand, schrieben viele Ärzte Cannabis medizinische Wirkungen zu. So führte zum Beispiel William B. O’Shaughnessy Cannabis erstmals als Behandlung gegen Tetanus und andere konvulsive Erkrankungen ein [3]. Ungefähr zur gleichen Zeit experimentierte der französische Arzt Jean-Jacques Moreau de Tours mit der Anwendung von Cannabis–Präparationen in der Behandlung psychischer Erkrankungen [4]. Bald danach, im Jahr 1851, wurde Cannabis in die 3. Edition der *Pharmacopoeia of the United States (USP) *aufgenommen. In mehreren Revisionen der USP wurde detailliert beschrieben, wie man Extrakte und Tinkturen getrockneter Cannabisblüten herstellt, um sie als Analgetika, Hypnotika und Antikonvulsiva zu verwenden [1, 5]. Wachsende Sorge hinsichtlich Abhängigkeit und psychoaktiver Wirkung von Cannabis führte Anfang des 20. Jahrhunderts zu einem Verbot von Cannabis in einigen Bundesstaaten der USA und 1937 mit dem „Marihuana Tax Act“ zu einem kompletten Verbot in den USA. 1942 entfernte die American Medical Association Cannabis aus der 12. Ausgabe der *U. S. Pharmacopoeia*. In Österreich wurde Cannabis 1963 infolge internationaler Abkommen verboten.

### Die Cannabispflanze

Kultiviertes Cannabis wird dem Genus *Cannabis*, Familie Cannabinacae, zugeordnet [6]. Auf Basis genetischer Varianten wurde von mehreren Forschern ein multitypisches Genus, mit zumindest zwei Spezies, *Cannabis sativa* und* Cannabis indica*, vorgeschlagen [7, 8]. Andere Forscher schlugen eine einzige Species *Cannabis sativa* vor, wobei die genetischen Unterschiede durch Unterschiede auf Ebene der Subspezies, sowie auf Ebene der Varietäten angenommen wurden [9].

### Die chemischen Bestandteile von Cannabis

Bis heute wurden mehr als 400 Pflanzenkomponenten, darunter 104 verschiedene Cannabinoide im Cannabis identifiziert. Cannabinoide sind eine Gruppe psychoaktiver, chemischer Bestandteile, die mit Cannabinoid-Rezeptoren u. a. im menschlichen Gehirn interagieren ([10]; Tab. 1).

**Table 1:** 

Delta-9-Tetrahydrocannabinol (THC)	Psychoaktiv, schmerzlindernd, aufheiternd, muskelentspannend, appetitanregend
Cannabinol (CBN)	Leicht psychoaktiv, Oxidationsprodukt von THC, beruhigend, antibakteriell, kann den Augeninnendruck senken
Tetrahydrocannabivarin (THCV)	Leicht psychoaktiv, ähnelt THC, appetitzügelnd
Cannabidiol (CBD)	Nicht psychoaktiv, psychoseprophylaktisch, mögliche Einsatzgebiete: Epilepsie, Schlafstörungen, Bewegungsstörungen, Psychosen
9‑Tetrahydrocannabinolsäure (THCA)	Nicht psychoaktiv, mit THC verwand, entzündungshemmend, appetitanregend
Cannabichromen (CBC)	Nicht psychoaktiv, zweithäufigstes Cannabinoid, kann die Wirkung von THC unterstützen, Schmerzlindernd, beruhigend, stimmungsaufhellend
Cannabidivarin (CBDV)	Nicht psychoaktiv, Homolog von CBD, antiepileptisch, krampflösend
Cannabigerol (CBG)	Nicht psychoaktiv, Phytocannabinoid, im Nutzhanf, entzündungshemmend, schmerzlindernd

Tetrahydrocannabinol (THC) wird in den glandulären Trichomen in Blüten, Blättern und Deckblättern der weiblichen Pflanze exprimiert und hat den stärksten psychoaktiven Effekt. Ein weiterer wichtiger Bestandteil von *Cannabis sativa ist das *Cannabidiol (CBD), das am häufigsten in Hanf vorkommende Cannabinoid. THC und CBD haben einen gemeinsamen Vorläufer, die *Olivetolsäure.*

Die am meisten beforschten Cannabinoide sind THC, CBD und Cannabinol (CBN).

In Hinblick auf Abhängigkeitserkrankungen sind vor allem die Arten Cannabis sativa und indica, sowie ihre diversen Kreuzungen von Bedeutung. Cannabis ruderalis spielt aufgrund des kaum vorhandenen THC-Anteils von unter 0,3 % nur eine untergeordnete Rolle.

Die Cannabispflanze ist diözisch, sprich getrenntgeschlechtlich. Es können weibliche und männliche Pflanzen unterschieden werden, die weiblichen Pflanzen weisen einen deutlich höheren THC-Gehalt auf. Daher werden von Konsumenten primär weibliche Pflanzen verwendet.

Die THC-Konzentrationen wurde durch entsprechende Züchtungen und Anbaumethoden in den vergangenen Jahrzehnten erhöht [11].

### THC – Delta-9-Tetrahydrocannabinol

Die Wirkung von THC ist meist aufheiternd, muskelentspannend, antiepileptisch, brechreizhemmend, appetitsteigernd, entzündungshemmend, fiebersenkend, Augeninnendruck-senkend, bronchodilatatorisch, beruhigend und schmerzhemmend. Diese Wirkungen lassen die möglichen Anwendungsgebiete in der Medizin vermuten, welche zum Teil auch in Erprobung sind.

Beim Konsum von THC-haltigem Cannabis kommt es, je nach Einnahmeart, Dosierung, aktueller psychischer Verfassung und dem individuellen Ansprechen zu einem, primär von THC verursachten, Rauschzustand.

Bei Inhalation setzt die Wirkung schon nach einigen Minuten ein, nach rund 15 min wird die Hauptwirkung erreicht, die bis zu vier Stunden anhält. Bei der oralen Anwendung (z. B. in Form von Cookies) dauert es hingegen 30–90 min bis zur ersten spürbaren Wirkung, welche dann nach zwei bis drei Stunden ihr Maximum erreicht und danach im Zeitraum von vier bis acht Stunden wieder abnimmt [12].

Es kommt zu einem sedierten bis euphorischen Zustand mit einer vom Konsumenten intensiviert empfundenen Wahrnehmung (entaktogene Wirkung). Die Euphorie kann aber auch (vor allem bei hohen Dosierungen) in Missstimmung, Depression, Angst und panikartige Erlebniszustände mit dem Gefühl von Kontrollverlust umschlagen. Weiters zeigen sich veränderte Zeitwahrnehmung, dissoziative Phänomene bis hin zu Halluzinationen und paranoide Gedanken. Die Reaktionsfähigkeit ist beeinträchtigt. Aufmerksamkeit, Konzentration, Feinmotorik und mnestische Fähigkeiten sind herabgesetzt.

Auf somatischer Ebene kann es zu Schwindel, einer verwaschenen Sprache, Müdigkeit, Mundtrockenheit, Übelkeit, Kopfschmerzen, reduziertem Tränenfluss, konjunktivalen Injektionen, Muskelrelaxation, gesteigertem Appetit, Tachykardie, Schwitzen, verschwommenem Sehen, orthostatischer Dysregulation und kardialer Ischämie kommen.

Todesfälle durch eine THC-Intoxikation sind, im Gegensatz zu Intoxikationen mit synthetischen Cannabinoiden, bisher nicht beschrieben. Es traten selbst bei Höchstdosen im Tierexperiment mit Affen bei 9000 mg/kgKG oral verabreichtem THC keine Todesfälle auf [13].

Im Rahmen des Langzeitgebrauchs kann es bei der Anwendung von Cannabis auch zu einer Toleranzentwicklung kommen, diese betrifft überwiegend die Wirkung auf die Psyche, Beeinträchtigungen der Psychomotorik aber auch die Wirkung auf das Herz-Kreislaufsystem. Ein Gewöhnungseffekt wird auch auf Rezeptorebene im Endocannabinoidsystem vermutet.

Langzeitnebenwirkungen betreffen vor allem die Psyche und das Denken, sowie das Immun- und Hormonsystem, wobei die Wirkungen auf das Immun- und Hormonsystem eher als gering einzuschätzen sind [14].

Gesundheitliche Auswirkungen hängen auch von der Art der Anwendung ab. So ist z. B. das Konsumieren einer Cannabis-Zigarette (Joint) durch die tiefe Inhalation mit einer wesentlich höheren Belastung an kanzerogenen Inhaltsstoffen durch den Verbrennungsprozess verbunden als bei konventionellem Tabakkonsum. Konsumenten verwenden auch andere Applikationsformen, wie z. B. das Vaporisieren. Die dabei freigesetzten THC-Mengen sind weitaus größer als beim Verbrennen in Cannabis-Zigaretten.

### CBD – Cannabidiol

Cannabidiol (CBD) kommt in großen Mengen vor allem im Faserhanf (cannabis ruderalis) vor. Der genaue Wirkmechanismus von Cannabidiol ist bisher noch nicht eindeutig geklärt. Es kann die schmerzlindernden Eigenschaften des THC verstärken. Es soll beruhigend, entzündungshemmend, antiepileptisch, anxiolytisch, antipsychotisch und Augeninnendruck-senkend wirken, hat jedoch keine dem THC vergleichbaren psychoaktiven Effekte. Die psychotropen Wirkungen werden jedoch erst bei vergleichsweise hohen Cannabidiolkonzentrationen erzielt [14].

Die Entwicklung der vergangenen Jahre zeigte einen rasant zunehmenden Gebrauch von stark cannabidiolhaltigen Cannabisprodukten, welche in Österreich an Erwachsene frei verkäuflich sind, da sie als Nahrungsergänzungsmittel klassifiziert wurden. Hier könnten sich, ob der sedierenden und entspannenden Effekte der Ruderal-Pflanzen mit hohem CBD-Anteil (THC-Gehalt unter 0,3 %), auch Möglichkeiten für eine Alternative zu stark THC-haltigen Cannabisprodukten ergeben. CBD wird versuchsweise auch im medizinischen Bereich eingesetzt, etwa bei onkologischen Patienten im Sinne einer Reduktion von Nebenwirkungen der Chemotherapie, supportiv bei bestimmten Epilepsieformen, anderen neurologischen Erkrankungen und Schmerzerkrankungen.

Auch ein Zusammenhang zwischen hohem CBD-Gehalt und einem selteneren Auftreten von psychotischen Reaktionen konnte gezeigt werden [15].

### Marktlage

Laut österreichischem Drogenbericht [16] und dem EU-Drogenbericht [11] wird davon ausgegangen, dass der Großteil des in Österreich konsumierten Cannabis illegal importiert wird.

In den letzten Jahren ist eine Zunahme der Anzahl an Growshops und Headshops zu beobachten. Das Auftreten der Shops ist hier nicht nur beratend, sie liefern auch die Infrastruktur für den „Homegrow“ und treten sehr oft als Lobbyisten für eine Cannabis-Freigabe oder Legalisierung auf.

In Österreich beschlagnahmte Cannabismengen sind über die Jahre 2007 bis 2020 relativ konstant geblieben. Anzeigen und Verstöße gegen das Suchtgiftmittelgesetz bezogen auf Cannabis zeigen jedoch einen deutlichen Anstieg. Insgesamt machen mit Cannabis assoziierte Anzeigen mehr als vier Fünftel der Verstöße gegen das Suchtgiftmittelgesetz in Österreich aus [16].

Im Zuge der Corona-Pandemie kam es zu einer Zunahme von Eigenanbau durch den zwischenzeitlich erschwerten Schmuggel im Rahmen von Reisebeschränkungen. Die dahinterstehende illegale Industrie reagierte darauf sehr schnell, um Lieferengpässe zu vermeiden bzw. rasch zu beseitigen. Auch eine Zunahme des Online-Handels war zu beobachten [11].

Im Straßenhandel liegen die Preise für Cannabiskraut gegenwärtig um 10 € pro Gramm. Betreffend der Reinheit liegen die Schwankungen bei Cannabiskraut (Marihuana) zwischen 0,62 % und 33,49 % THC-Gehalt bei einem Median von 10,92 %, bei Cannabisharz zwischen 0,25 % und 52,31 % bei einem Median von 13,45 %, was eine auffallend hohe Schwankungsbreite darstellt [16]. Interessant dabei ist die durchschnittliche Zunahme des THC-Gehalts in den letzten zehn Jahren für Marihuana um ca. 50 % und für Haschisch um mehr als 150 % bei völlig gleichbleibenden Preisen [17].

### Nachweisbarkeit

Die Nachweisbarkeit von Cannabis ist abhängig vom gewählten Testverfahren, der individuellen Stoffwechsellage und dem Konsummuster. Im Folgenden werden mögliche Testvarianten mit ihren Vor- und Nachteilen kurz umrissen. In der Praxis werden zum Nachweis eines Cannabis-Konsums meist Harntests eingesetzt.

Der **Nachweis im Urin** wird primär über Abbauprodukte von THC durchgeführt, die innerhalb weniger Stunden nach dem Konsum entstehen. Die Konzentration dieser Verbindungen steigt im Harn über eine längere Zeitspanne an und ist hier auch wesentlich länger nachweisbar als im Blut. Die Harnkontrolle sollte unbedingt unter Sicht und in Einrichtungen erfolgen, die Erfahrung in der Testung haben. Um eine Verfälschung der Laborergebnisse über Verdünnen des Harns zu entlarven, wird die Kreatininmenge im Harn mitbestimmt. Weitere Einflussfaktoren, wie Körperfettanteil, Konsummuster und körperliche Verfassung wirken sich auch auf die Nachweisbarkeitszeit aus. So kann die Nachweisbarkeit bei Einmal‑/Gelegenheitskonsum einige Tage betragen, bei Dauerkonsum in Extremfällen bis zu drei Monate nach Ende des Konsums [18]. Zu beachten ist jedoch, dass mit den herkömmlichen Harntests nur das THC, jedoch nicht synthetische Cannabinoide nachgewiesen werden können. Für letztere bedarf es eigener Testkits. Deren Verwendung wäre insbesondere in sensiblen Bereichen, wie der Justiz, bei gerichtlichen Auflagen oder im Bereich der Straßenverkehrsordnung zu empfehlen. Auch bei unklaren Intoxikationssymptomen könnten synthetische Cannabinoide eine Rolle spielen.

Bei der **Blutanalyse** wird in der Regel die Menge des THC und teilweise auch der Gehalt der Abbauprodukte THC-Carbonsäure und 11-Hydroxy-THC ermittelt. Bei der Inhalation erreicht der THC-Gehalt des Blutes schon nach wenigen Minuten seinen maximalen Wert und sinkt dann relativ schnell wieder, in der Regel schon nach einer Stunde, auf Werte unterhalb von 20 Nanogramm pro Milliliter Serum ab. Nach zwei bis drei Stunden sind, je nach Stärke und Regelmäßigkeit des Konsums, meist nur noch Werte unterhalb von 10 Nanogramm THC pro ml Serum nachweisbar. Bei oraler Einnahme von Cannabisprodukten wird die maximale THC-Konzentration im Blut verzögert nach etwa einer Stunde erreicht und der Spiegel kann bis zu sechs Stunden gleichbleiben. Der maximale Wert ist allerdings geringer als bei der Inhalation von Cannabisprodukten [14].

**Speichel oder Schweißtests** funktionieren meist nach einem Immunoassay-Verfahren und eignen sich vor allem für die Überprüfung einer Verdachtssituation, haben allerdings deutliche Schwankungen in ihrer Spezifität und Sensitivität. Weiters sind die Ergebnisse von der korrekten Anwendung abhängig. Die Nachweisbarkeit liegt bei den meisten Tests bei 12 bis 24 h nach dem letzten Konsum. Insgesamt handelt es sich um eine unzuverlässige Testmethode [14].

**Haaranalysen** eignen sich nicht für die Beurteilung einer akuten Intoxikation, sie erlauben vielmehr eine Aussage, je nach Länge des Haars, über den Konsum im längeren zeitlichen Verlauf. Dies erlaubt auch eine Überprüfung zum Beispiel im Längsverlauf einer gerichtlichen Auflage. Chemische Schädigungen der Haarwurzel oder der Haare, wie es bei bestimmten Friseuranwendungen üblich ist, beeinflussen die Testung oder machen sie unbrauchbar [14].

## Das Endocannabinoid-System

Ähnlich wie bei Opioiden ist der Mensch sowie die meisten anderen Lebewesen von Strukturen des körpereigenen Cannabinoid-Systems, des sog. „Endocannabinoid-Systems“ (eCB) durchdrungen. Dieses System umfasst im Wesentlichen 2 Rezeptoren (CB1‑R und CB2-R) und Substanzen, die daran binden können („Liganden“, also die Cannabinoide) sowie Strukturen, die nach Bindung eines Liganden an die Cannabinoid-Rezeptoren einen nachgeschalteten Effekt vermitteln.

Anfang der 1990er-Jahre wurden die bisher beschriebenen Cannabinoid-Rezeptoren „CB1-R“ und „CB2-R“ entdeckt [19, 20]. CB1‑R und CB2‑R sind im präsynaptischen Bereich von inhibitorischen, aber auch exzitatorischen Synapsen im ZNS sowie in der Peripherie lokalisiert.

CB1‑R sind vorwiegend an Zellen des zentralen Nervensystems lokalisiert, in besonders hoher Dichte im Kleinhirn, in den Basalganglien, der Substantia nigra, dem Riechkolben sowie im Hippocampus. CB1‑R finden sich auch an Zellen des peripheren Nervensystems, wie z. B. im Darm. Sie sind nach heutigem Wissensstand Teil funktioneller Netzwerke, die an höheren Gehirnfunktionen wie Vergnügen, Freude, Wohlbefinden, Gedächtnis, Gedankenstruktur, Konzentration, sensorischen Empfindungen, Zeitempfinden sowie motorischer Koordination beteiligt sind [21, 22].

CB2‑R hingegen sind vor allem in peripheren Bindegeweben (hier vor allem in Strukturen des Knochenauf- und abbaus) sowie auf Zellen des Immunsystems, wie auch intrazerebral in der Mikroglia, lokalisiert. Funktionell spielen CB2‑R wahrscheinlich eine Rolle in antiinflammatorischen sowie immunmodulierenden Prozessen [23, 24].

Eine Aktivierung von Cannabinoid-Rezeptoren löst eine Kaskade unterschiedlicher Effekte aus, die sich zum Teil untereinander beeinflussen [21, 25]. Akute somatische Effekte sind unter anderem Tachykardie, erhöhter Blutdruck, Anstieg der Herzfrequenz sowie trockene Schleimhäute im Mund- und Rachenbereich. Akute psychische Effekte können Euphorie, angehobene Stimmung, verändertes und intensiveres Wahrnehmungsempfinden, Reizbarkeit sowie Koordinationsschwierigkeiten sein. Manche Cannabinoide scheinen diese Wirkungen zu beeinflussen und teilweise zu antagonisieren, sodass sie primär spannungslösend wirken und keine wahrnehmungsverändernden Eigenschaften haben. CBD hat einen solchen Effekt, und bindet dabei offenbar nicht direkt an CB1- oder CB2-Rezeptoren, sondern scheint seine Wirkung über einen bisher unbekannten Mechanismus zu entfalten. Zusätzlich wurde bei bestimmten Endocannabinoiden eine antiemetische, antiinflammatorische sowie analgetische Wirkung beschrieben. Das Cannabinoid-System könnte eine Rolle in der Erregbarkeit von Nervenzellen spielen, und bestimmte Cannabinoide daher antiepileptische Potenz besitzen.

Bisher wurden über 100 verschiedenen Endocannabinoide identifiziert, die in Cannabis-Produkten in völlig unterschiedlicher Zusammensetzung und Konzentration vorkommen können. Durch industrielle Züchtung wird daher versucht, Einfluss auf diese Zusammensetzung zu nehmen, und bestimmte Eigenschaften zu verstärken.

Abschließend sei noch auf eine weitere wesentliche Funktion des eCB hingewiesen. Das körpereigene eCB spielt schon in der frühen Embryonalphase eine wichtige Rolle für viele physiologische Hirnreifungsprozesse. Die Funktion des eCB ist in der Folge wechselnd stark in seiner Ausprägung und beeinflusst bis zum Abschluss der Gehirnreifung am Ende der Adoleszenz wesentliche Reifungsprozesse, die sich in der Zeit der sexuellen Reife besonders stark auswirken [26]. Es zeigt sich in immer mehr tierexperimentellen Befunden, die zunehmend auch Bestätigung beim Menschen finden, dass insbesondere der intensive Cannabiskonsum eine deutliche Beeinflussung dieser Prozesse in der Schlüsselphase der Gehirnentwicklung nach sich ziehen und damit besonders schädliche Auswirkungen hervorrufen kann. Trotz wesentlicher Unterschiede geben tierexperimentelle Untersuchungen klare Hinweise darauf, dass das eCB eine wichtige Rolle bei der abschließenden Myelinisierung der Nervenfasern, beim synaptischen und axonalen Abbau in der Pubertät und bei der Ausreifung verschiedener Neurotransmittersysteme im Gehirn spielt. Somit kann der frühe und regelmäßige Cannabiskonsum irreversible Schäden bei der Gehirnentwicklung verursachen [26, 27].

## Epidemiologische Daten

Zum Gebrauch von psychotropen Substanzen für die Altersgruppen ab dem 13. Lebensjahr gibt es in Österreich eine gute Datenlage, bedingt durch die Einbindung in europäische und andere internationale Studien. Hier sind vor allem die HBSC Studie (Health Behaviour in School-aged Children) und die ESPAD (European *School* Survey Project on Alcohol and other Drugs) zu nennen.

Bei den HBSC-Studien (u. a. [28]) werden regelmäßig die Gesundheit und gesundheits- und psychosoziale Parameter bzgl. Verhalten, Einstellung und Wahrnehmung bei Schülern der 5., 7. und 9. Schulstufe abgefragt, um entsprechende Präventionsangebote zu kreieren. Die 13- und 15-jährigen Schüler werden dabei auch nach ihren verschiedenen Konsummustern, unter anderem auch zu Nikotin, Alkohol und Cannabis befragt.

Die ESPAD-Studie (u. a. [29]) wurde im Jahre 2019 in Österreich zum 4. Mal nach 2003, 2007 und 2015 mittels Onlineerhebung durchgeführt. 10297 Schülerinnen und Schüler der 9. und 10. Schulstufe aus allen Schultypen wurden hier zu ihren Konsummustern befragt.

Weitere wichtige Befragungen sind das „Wiener Suchtmittelmonitoring“, bei dem im 2‑Jahres Abstand ca. 600 Personen wesentlicher Altersstufen in persönlichen Interviews befragt werden, das „Drogenmonitoring Oberösterreich 2019“ [30] und die Repräsentativerhebungen zu Konsum und Verhaltensweisen mit Suchtpotential (u. a. [31]) in 4‑jährigem Abstand, in welchen ebenso persönliche Interviews zur Anwendung kommen. Weitere Datenquellen sind Publikationen von Amtsärzten, von Krankenanstalten über gemeldete Behandlungen, sowie die Anzeigenstatistik der Polizeibehörden.

Bei den großen Befragungen kommen im Wesentlichen vordefinierte Parameter zur Abfrage. Die Lebenszeitprävalenz fragt nach jemals erfolgtem Konsum einer Substanz in der bisher erfolgten Lebensspanne und beschreibt, wieviel Menschen der jeweiligen Alterskohorte je Kontakt und zumindest Probierkonsum hatten. Diese Angaben sind auch nach der Schwelle bzw. Verfügbarkeit für unterschiedliche Substanzen zu interpretieren.

Der Konsum in den letzten 12 Monaten, die Jahresprävalenz, gibt Informationen über zumindest moderaten Konsum und etwaige Lebensbereiche mit Einstiegs- und Ausstiegsphasen.

Das Konsummuster in den letzten 30 Tagen verweist auf aktuellen Konsum, wobei hier in den Studien teilweise noch nach genaueren Konsummustern gefragt wird. Dabei ist jedoch zu berücksichtigen, dass gerade beim regelmäßigen und hochfrequenten Cannabiskonsum (6-mal oder häufiger pro Monat) auf Grund der geringen Anzahl der Konsumenten nicht wirklich eine Aussage-Relevanz besteht. Die anderen Untersuchungsbereiche weisen jedoch eher stabile Größen aus.

Die Lebenszeitprävalenz ergibt je nach Studie und dem abgefragten Altersrange relativ ähnliche Zahlen in leicht ansteigender Häufigkeit mit steigendem Alter. Diese liegt im Mittel bei ca. 20 % für eine repräsentative Gesamtbevölkerung im Alter zwischen 15 und 69 Jahren. Andere Autoren [32] konstatieren, dass aufgrund der Annahme einer allgemeinen Dunkelziffer die Anzahl derer, die schon jemals Cannabis konsumiert haben, zwischen 33 und 50 % der österreichischen Bevölkerung liegt.

Wenn aber nur die Jugendlichen und jungen Erwachsenen (15–24 Jahre) betrachtet werden, zeigen sich Prävalenzraten von bis zu 42,3 %. Die abgefragten Alterskohorten umfassen allerdings oftmals 5 bis 10 Jahre und vermitteln den Durchschnitt einer entwicklungsmäßig sehr inhomogenen Gruppe, wobei die jüngste Altersgruppe in den ESPAD Studien (ab 13 Jahren) befragt worden ist. Insgesamt weisen die Zahlen aus, dass auch Menschen zwischen dem 20. und 30. Lebensjahr noch vermehrt zum ersten Mal konsumieren. **Dies zeigt, dass Cannabis in Österreich und auch in den wesentlichen Industriestaaten, die am häufigsten konsumierte illegalisierte Droge ist.**

Die Jahresprävalenz für Jugendliche liegt zwischen 19 % in der ESPAD-Studie und 23,2 % im Drogenmonitoring OÖ, wobei in den letzten 15 Jahren doch insbesondere bei den männlichen Konsumenten eine deutliche Zunahme um bis zu 25 % [28] zu bemerken ist, die Zunahme bei den weiblichen Konsumentinnen fällt nur leicht aus.

Die Monatsprävalenz liegt hingegen bei den meisten Untersuchungen aus dem skizierten Pool an Studien stabil bei ca. 11 %, wobei auch hier, je nach Altersrange eine leichte Steigerung über die letzten Jahre zu sehen ist. Von den Konsumenten, die in den letzten 30 Tagen konsumiert haben, gab jeder Dritte (4,0 %) einen regelmäßigen Konsum (6 × oder mehr im letzten Monat) und jeder Siebte (1,5 %) einen hochfrequenten Konsum (mindestens 20 × im letzten Monat) an [29]. Der dabei ermittelte Prozentsatz an riskant konsumierenden Jugendlichen ist aber auf Grund der vermutlich systematischen Überschätzung eher als Obergrenze zu werten und mit großer Wahrscheinlichkeit erheblich über der realen Anzahl angesiedelt. Bei den differenzierteren Analysen der Autoren des österreichischen Teils zeigen ca. 1 % der Befragten ein starkes Risikoverhalten.

In der rezenten ESPAD-Studie von 2019 wird konstatiert, dass der Einstieg in den Cannabiskonsum bis zum 13. Lebensjahr in einem sehr beschränkten Ausmaß erfolgt. Dann vollzieht sich aber ein kontinuierlicher Anstieg mit bis zu 30 % Lebenszeiterfahrung bei den 16-Jährigen. Das entspricht auch etwa der Schwarzmarkt-Verfügbarkeit von Cannabis. Danach befragt, wie einfach es sei, an Cannabis zu gelangen, gaben 30 % der 14-jährigen und 51 % der 17-jährigen an, dass es leicht für sie sei [29].

Im Vergleich dazu gaben in dieser Studie [33] die Jugendlichen an, dass in den letzten 30 Tagen ein Alkoholkonsum bei ungefähr 2/3 der Gesamtstichprobe erfolgt sei. Ca. 5 % berichten über regelmäßigen oder auch hochdosierten Alkoholkonsum, welcher längerfristig als bedenklich einzuschätzen ist. Für Nikotin zeigen sich Zahlen von ca. 20 % für regelmäßigen, d. h. täglichen Konsum und von 29 % für einen Konsum in den letzten 30 Tagen. Hier ist das Geschlechterverhältnis ausgeglichen.

Zusammenfassend zeigt sich, dass Bevölkerungsbefragungen eher grobe quantitative Hinweise geben, welche Menschen grundsätzlich Konsumerfahrung (Lebenszeitprävalenz) haben. Wie viele Menschen welcher Alterskohorte jedoch aktuell Cannabis und in welcher Frequenz konsumieren ist schon bedeutend schwieriger zu erfassen, insbesondere der risikoreiche Konsum, da die hier erhobenen Zahlen eher als Obergrenze zu verstehen sind. Hierzu geben Behandlungsdaten bessere Auskunft, insbesondere dürften Personen mit behandlungsbedürftigem Cannabiskonsum die zahlenmäßig zweitgrößte Gruppe sein (nach solchen mit Alkoholkonsum), die 10 % aller Behandlungen von Abhängigkeitserkrankungen pro Jahr ausmachen. Dies sind etwa 2000 Personen bzw. 0,03 % der österreichischen Bevölkerung ab dem 15. Lebensjahr [34].

## Definition der Cannabis-assoziierten Störungen in ICD und DSM, Konsummuster

Das DSM‑V definiert „Störungen durch Cannabiskonsum“ als Störung im Zusammenhang mit den Erzeugnissen der Cannabispflanze in all ihren Varianten, einschließlich der synthetischen Cannabinoide mit unterschiedlich ausgeprägter Wirkung an den CB1‑R und CB2‑R.

Im DSM‑V [35] wird die Störung diagnostisch über die Auswirkungen des Konsums definiert: Kontrollverlust bezüglich Menge und Konsumdauer, erfolglose Versuche, den Konsum zu beenden, starkes Craving, berufliche, soziale oder zwischenmenschliche Probleme als Folge des Konsums (genaue Kriterien siehe DSM-V). Die genannten Kriterien sind allerdings nicht eindeutig und objektiv überprüfbar, sodass die Entscheidung, ob ein Kriterium erfüllt wird, von der subjektiven Einschätzung des Untersuchers abhängt. Dies ist insofern von Bedeutung, da nach DSM‑V die Schweregradeinteilung davon abhängt, wie viele Kriterien erfüllt werden. Als bedeutsam wird im DSM‑V das Auftreten eines Cannabisentzugssyndroms beschrieben. Toleranz und Entzug als Merkmal der Störung wird aber bei medizinisch indiziertem Konsum nicht berücksichtigt. Auch im DSM‑V wird auf die Häufigkeit komorbider Störungen und den häufigen Versuch der Selbstbehandlung hingewiesen.

Der „schwere oder langdauernde Cannabiskonsum“ wird im DSM‑V in den Kriterien für das Cannabisentzugssyndrom näher eingeengt als beinahe täglicher Konsum über mehrere Monate. Beschrieben wird im DSM‑V auch der „unproblematische Cannabiskonsum“, ebenfalls mit sehr unklaren Kriterien.

Der ICD-10 [36] definiert „Psychische Verhaltensstörungen durch Cannabinoide“ in verschiedenen klinischen Erscheinungsbildern. Diese Zustandsbilder unterliegen klaren psychopathologisch definierten Kriterien bei nachgewiesenem Cannabinoidkonsum. Dabei spielen Zeitkriterien und die (negativen) Auswirkungen des Konsums v. a. auf zwischenmenschliche Beziehungen eine wichtige Rolle.

Im Abschnitt (F1x.xx) des ICD-10 wird versucht, diverse Substanzen und deren psychische Verhaltensstörungen zu beschreiben. Kritisch anzumerken ist die deutliche Fokussierung dieses Kapitels auf Erwachsene, da weder die Auswirkungen des Konsums auf die jeweiligen adoleszenten Entwicklungsphasen noch der Einfluss der jeweiligen Entwicklungsphasen auf die unterschiedlichen Konsummuster berücksichtigt werden. Diverse Cannabinoid-Zubereitungen oder Konsumformen finden ebenso keine Erwähnung.

Der ICD-11 [36] wurde im Mai 2019 von der 72. Worth Health Assembly verabschiedet und ist seit 01.01.2022 gültig. Es gilt eine Übergangsfrist von 5 Jahren, dann sollen ausschließlich ICD-11 Codes angewandt werden. Der Zeitpunkt der Implementierung im deutschsprachigen Raum ist noch unklar, aktuell ist der Übersetzungsprozess im Gange. Psychische Störungen („mental, behavioral or neurodevelopmental disorders“) werden im Abschnitt 6 des ICD-11 codiert, Suchterkrankungen im Kapitel 6C „Störungen aufgrund von Substanzgebrauch oder Suchtverhalten“ („Disorders due to substance use or addictive behaviours“). Umstritten ist hier die Zusammenführung von „Substanzgebrauchsstörung und Suchtverhaltensstörung“, wobei bei letzterem nur gaming/gambling disorder angeführt wird.

Störungen mit Cannabis werden unter 6C41 codiert, Störungen verbunden mit synthetischen Cannabinoiden unter 6C42. Cannabis wird klassifiziert nach Präparation, Art und Applikation, mit dem zentralen psychoaktiven Cannabinoid Delta-9-tetrahydrocannabinol (THC) und den entsprechenden psychischen Effekten. Unter „Synthetische Cannabinoide“ werden hunderte chemisch synthetisierte Stoffe mit agonistischer Wirkung an den endogenen Cannabinoid-Rezeptoren mit entsprechender klinischer Wirkung beschrieben. Aufrechterhalten wird die Unterscheidung von „problematischem und schädlichem“ Konsum bzw. ein- oder mehrmaligem Konsum mit den zentralen Kriterien der Gefahr der Vergiftung, der Schädigung und Abhängigkeit. Die Kriterien für „Abhängigkeit“ sind Kontrollverlust, starker innerer Drang und täglicher oder fast täglicher Konsum für zumindest 3 Monate bzw. Symptome von Abhängigkeit über 12 Monate. Diese Kriterien lassen weiterhin breiten Interpretationsspielraum zu.

Das ICD-11 übernimmt somit nicht die Abstufungen der Substanzgebrauchsstörung des DSM‑V. In der Kategorie Abhängigkeit können die „rasche und volle Remission“ (early full remission), die „Teilremission“ (sustained partial remission) und die „stabile Remission“ (sustained full remission) codiert werden. Spezielle Kriterien zum Konsum im Jugendalter existieren nicht. Das ICD-11 stellt somit eine Erweiterung des ICD-10 dar, z. B. um die Gruppe der synthetischen Cannabinoide und ermöglicht differenziertere Codierungsmöglichkeiten, wie z. B. Arten der Abstinenz.

*Wie auch die unklaren Definitionen von „schädlichem“ Konsum im ICD-10, im ICD-11 sowie* auch im DSM‑V zeigen, gibt es derzeit keinen Konsens darüber, wann Cannabis-Konsum als problematisch zu betrachten ist. In der Literatur spricht man von „heavy use“, „risky use“, „problematic use“, „dependence“ oder „dangerous use“. In vielen Studien werden jeweils unterschiedliche Kriterien zur Beurteilung verwendet und allgemein gültige Kriterien konnten daher bis dato nicht etabliert werden [37].

## Neurobiologische Grundlagen und Folgen von Cannabiskonsum – ein Überblick

Durch Endocannabinoid vermittelte Prozesse sind signifikant an der Entwicklung des Säugetiergehirns beteiligt [38, 39]. Besonders das adoleszente Gehirn kann leicht Störungen im körpereigenen eCB entwickeln, die mit Änderungen der Hirnentwicklung sowie der Hirnplastizität und entsprechenden verändertem Verhalten/Kognition einhergehen [40, 41].

CB1-Rezeptoren und Endocannabinoide modulieren im Gehirn u. a. die Aktivität von Neuronen und der Oligodendrozyten. Letztere sind für die Isolierung der Axone von Nervenzellen verantwortlich und in der Folge auch für deren Versorgungsmechanismus. Die Myelinisierung insbesondere in Assoziationsbereichen im Gehirn, im Kleinhirnbereich und in der Formatio reticularis ist in der 2. und 3. Dekade besonders ausgeprägt. Dies ist die Zeit, in welcher viele Jugendliche Cannabis konsumieren. Es zeigt sich zwar keine Unterscheidung zwischen Nicht-Konsumenten und Konsumenten bei der Mikrostruktur der weißen Substanz, jedoch ist mittlerweile bekannt, dass früher und zumindest wöchentlicher Konsum von Cannabis in Zusammenhang mit funktionellen Aspekten in bestimmten Bereichen des rechten unteren Fasciculus longitudinalis und im Fasciculus uncinatus steht. Diese Erkenntnisse vermitteln das Risiko einer besonderen Vulnerabilität der Hirnreifung während der Adoleszenz bei Cannabiskonsumenten [42].

Oftmals sind die Dosisunterschiede bezüglich der jeweiligen Folgen sehr gering. Cannabinoid-Agonisten im Gehirn wirken toxisch auf die Nervenzellen und können je nach zugeführter Dosis eine Myelinisierung oder eine Demyelinisierung der Axone bewirken. Ebenso kann das Erleben von chronisch-psychosozialem Stress bei Konsumenten die Vulnerabilität erhöhen, sodass für die Myelinbildung verantwortliche Gene durch Cannabinoid-Agonisten in ihrer Expression reduziert werden. Der präfrontale Kortex, das Corpus callosum und andere Faserstrukturen scheinen hier besonders betroffen zu sein. Ebenso finden sich epigenetische Effekte von Cannabinoiden, die die Hirnentwicklung beeinträchtigen können [43].

Aus der tierexperimentellen Grundlagenforschung zum eCB gibt es zahlreiche Befunde, die eine Beeinträchtigung der Kognition, insbesondere des Lernens und des Gedächtnisses durch externe Zuführung von Cannabisprodukten zeigen (u. a. [44]). Diese Beeinträchtigungen bleiben auch bestehen, nachdem die Exposition beendet worden ist, wobei das Ausmaß der Beeinträchtigung sehr stark von der jeweiligen Entwicklungsphase des Tieres abhängig ist [26]. Auch wenn die Ergebnisse zum Teil mit großer Zurückhaltung und Vorsicht auf die menschliche Spezies zu übertragen sind, zeigen sich doch immer mehr Hinweise auf funktionelle wie hirnmorphologische Folgen von Cannabiskonsum in klinischen und post mortem-Studien.

Eva Hoch und ihr Team haben angesichts der Debatte um die (un-) bedingte Freigabe von Cannabis in Deutschland eine umfassende Arbeit über die zahlreichen wissenschaftlichen Veröffentlichungen der letzten Jahre erstellt [45]. Dabei werden die unterschiedlichen Ergebnisse der menschlichen Kognition ausdifferenziert nach verschiedenen Bereichen, wie dem Gedächtnis, der Aufmerksamkeit, den Exekutivfunktionen, der Psychomotorik, der Entscheidungsfähigkeit und der Intelligenz. Diese Bereiche steuern eine Vielzahl bewusster und unbewusster neuronaler Prozesse des Gehirns, die an der Verarbeitung externer und interner Information beteiligt sind. Das eCB spielt dabei eine wichtige Rolle, u. a. bei der Modulation von hemmenden und erregenden Signalübertragungsprozessen im Gehirn [46]. Zusätzlich findet sich eine hohe Anzahl an CB1‑R im Frontalcortex, Hippocampus und Globus pallidus, jenen Hirnregionen, die mit besonderen kognitiven Funktionen in Verbindung gebracht werden [22].

Bei der **Gedächtnisleistung** unterscheidet man ein Arbeits‑, Kurz- und Langzeitgedächtnis, sowie das Erlernen und Wiedererkennen von Informationen, die auch noch gemäß der, die Information transportierenden Sinneskanäle in visuell oder auditiv unterteilt werden können.

Akuter Cannabiskonsum führt zu nahezu einheitlichen Defiziten beim Abrufen von Gedächtnisinhalten und in der Wiedererkennungsleistung [47], wobei diese Störungen bei Gelegenheitskonsumenten offensichtlich stärker ausgeprägt sind als bei chronischen Konsumenten. Die Untersuchungen zur Störung des Arbeitsgedächtnisses sind sehr heterogen und zeigen keine einheitlichen Ergebnisse.

Deutlich einheitlicher sind die Ergebnisse bei chronischem Konsum, die eine durchgängige Beeinträchtigung von Lernen und Gedächtnis in einer Vielzahl von Studien zeigen. Wird dann eine Abstinenz erreicht, verbessert sich die kognitive Leistungsfähigkeit mit deren Dauer. Die Ergebnisse zur Persistenz von Gedächtnisstörungen nach Abstinenz sprechen tendenziell für eine Verbesserung bis zu einer Remission der Störung der Gedächtnisleistung, wobei in den Studien oft nur kurze Beobachtungszeiträume von bis zu 6 Wochen beurteilt wurden [47].

Die **Aufmerksamkeit **wird in selektive, geteilte und andauernde Aufmerksamkeit unterteilt. Die Testungen erfordern eine längerfristige Konzentrationsfähigkeit und adäquate Reaktionen unter verschiedenen Rahmenbedingungen, wie ruhige Atmosphäre versus viele Ablenkungsmöglichkeiten.

Der akute Konsum von Cannabis zeigt hier durchwegs eine deutliche Beeinträchtigung sowohl aller Aufmerksamkeitsleistungen als auch der Dauer der Konzentrationsfähigkeit, wobei einige Befunde auf eine dosisabhängige Wirkung hinweisen [47].

Noch klarer findet sich die Einschränkung der Dauer der Aufmerksamkeitsleistungen bei chronischem Konsum. Nach Beendigung des Cannabiskonsums zeigt sich diese Beeinträchtigung in vielen Studien auch noch nach längerer Zeit, sodass hier ein langfristiger Residualeffekt vermutet wird [48].

Als **Exekutivfunktionen** werden Steuerungs- und Leitungsprozesse mit höheren übergeordneten kognitiven Funktionen beschrieben. Sie spielen eine zentrale Rolle bei der Regulation von Verhalten und Flexibilität, dem Problemlösen, der Inhibition und der Interferenzkontrolle, der Planung und Erledigung von Aufgaben.

Bei akutem Konsum erscheint die Datenlage eher inkonsistent. Broyd (2016) beschreibt vor allem eine Beeinträchtigung der Inhibitionsfähigkeit. Bei den anderen Parametern sind die Befunde noch deutlich widersprüchlicher.

Bei chronischem Konsum gibt es ebenso sehr widersprüchliche Ergebnisse, wobei verbleibende Störungen der Exekutivfunktionen vor allem bei älteren Probanden gesehen worden sind [47]. Diese konnten auch nach längerer Abstinenz, bis zu 12 Monaten, nachgewiesen werden. Jüngere Probanden zeigten hingegen keine Verbesserung. In einer anderen Meta-Analyse fanden sich deutliche Einschränkungen der Exekutivfunktionen auch nach 25tägiger Abstinenz [48].

Bewusst regulierbare, zielgerichtete und motorische Prozesse, welche Bewegungen, Haltung und Tonus einschließen werden als **Psychomotorische Funktionen** bezeichnet und sind im Alltagsleben, wie z. B. beim Autofahren oder beim Bedienen von Maschinen relevant. Hier zeigt der akute Konsum deutliche Beeinträchtigungen in einheitlicher Studienlage [47]. Beim chronischen Konsum findet sich eine heterogene Studienlage, wobei sich nach Abstinenz im Wesentlichen keine bleibenden Störungen zeigen [47, 48].

Der **Entscheidungsfindung** liegt die Verarbeitung von unterschiedlichsten Informationen zu Grunde, die in eine Einleitung adäquater Reaktionen und der damit verbundenen Wahl zwischen Alternativen mündet. Dieser Prozess ist höchst komplex und von vielen Faktoren beeinflusst. Akuter THC-Konsum zeigt deutliche Auswirkungen auf die Entscheidungsfindung, und hier besonders auf die Störung der Belohnungssensitivität sowie auf die adäquate Steuerung des Risikoverhaltens [47]. Gleiches gilt für den chronischen Konsum, wo deutliche Defizite in der Entscheidungsfindung mit einer verminderten Verlustsensitivität bei gleichzeitig erhöhter Sensitivität gegenüber Gewinnen gezeigt wurden. Bei jugendlichen Konsumenten steigert ein langfristiger Konsum von Cannabis das Risikoverhalten auch nach längerer Abstinenz [49].

Wechsler (1944) bezeichnet die **Intelligenz **als die zusammengesetzte Fähigkeit eines Individuums zweckvoll zu handeln, vernünftig zu denken und sich mit seiner Umwelt wirkungsvoll auseinander zu setzen [50]. Der daraus abgeleitete IQ hat verschiedene Bereiche, wie den mathematischen, sprachlichen, technischen, musischen, sozialen und emotionalen IQ.

Zu den Auswirkungen von akutem Konsum auf die Intelligenz gibt es derzeit keine belastbaren Studien. Beim chronischen Konsum zeigt insbesondere die Studie von Meier [51] einen eindeutigen Zusammenhang zwischen Dauer und Intensität des Cannabiskonsums, dem Alter und dem Abfall des IQ’s im Vergleich zum individuellen Ausgangsniveau. Bei der als klassisch zu bezeichnenden Untersuchung, wurde eine Neuseeländische Geburtenkohorte von über 1000 Probanden mehrfach im Jugendalter und dann erneut mit 38 Jahren untersucht und der jeweilige Cannabiskonsum erhoben. Dabei zeigte sich, dass früher bzw. adoleszenter Cannabiskonsum zu einem erheblichen Risiko von stärkerem IQ-Defizit, im Durchschnitt bis zu 8 Punkten, führen kann. Dieser Befund hatte bei Adoleszenten auch nach einer langen Abstinenzzeit von über einem Jahr Bestand im Verhältnis zu Konsumenten, die erst nach dem 18. Lebensjahr mit einem Cannabiskonsum begonnen hatten. Ähnliche Ergebnisse wurde u. a. von Fried in der „Ottawa prenatal prospective Study“ erhoben [52].

Diese Befunde wurden auch in einer neueren Studie von Mokrysz (2016) bestätigt, wobei jedoch der Intelligenzverlust bei Korrektur von Alkohol‑, Drogen- und Tabakkonsum deutlich geringer ausgefallen ist [53]. Der Autor konstatiert, dass ein Cannabiskonsum mit 15 Jahren mit einem frühen Einstiegsalter und regelmäßigem und starkem Konsum mit einer Verminderung des IQ’s und der Schulleistung verbunden ist. Ein moderater Cannabiskonsum sei hier jedoch nicht leistungsmindernd aufgefallen.

Die Durchführung kognitiver Tests unter Untersuchungsbedingungen, wie SPECT, PET und fMRT zeigt **funktionelle Hirnveränderungen**. In einzelnen Studien bei akutem Konsum konnte eine Reduktion des regionalen zerebralen Blutflusses bei moderaten Konsumenten in Teilen des Kleinhirns und im Thalamus, bei chronischen Konsumenten im Frontallappen [54] nachgewiesen werden.

Zusammenfassend kann jedoch aufgrund der heterogenen Befunde konstatiert werden, dass sich offensichtlich die Hirnaktivität unter akutem Cannabiskonsum verändert, es bleibt aber noch unklar, ob die Hirnaktivität erhöht oder vermindert ist [45].

Bei chronischem Konsum scheint es gute Evidenz für eine erniedrigte Aktivität des präfrontalen Cortex bei der Durchführung unterschiedlicher kognitiver Aufgaben zu geben [55]. Dieser Befund wurde mehrfach bestätigt und funktionelle Veränderungen konsistent für alle untersuchten kognitiven Prozesse beschrieben.

In einer rezenten Studie wurden 799 Cannabis konsumierende Jugendliche im Alter zwischen 14 und 19 Jahren MRT-Untersuchungen unterzogen. Hier zeigte sich, dass die kortikale Dicke besonders im Bereich des präfrontalen Cortex, und dort besonders in Bereichen, die reich an Cannabinoidrezeptoren waren, mit der im Laufe des Lebens konsumierten Cannabismenge korreliert im Vergleich zu abstinenten Kontrollen signifikant dosisabhängig abgenommen hatte [56]. Weiteres wird auch in vielen anderen Studien eine spezifische Verminderung der Gehirnaktivität bei chronischen Konsumenten festgestellt.

Insgesamt jedoch sind die Ergebnisse aufgrund der unterschiedlich verwendeten Designs schwer zu vergleichen, auch über die Persistenz der Veränderungen lassen sich keine klaren Aussagen ableiten.

## Altersspezifische Effekte

Wurden früher bei den Studien kaum Differenzierungen der Cannabiskonsumenten nach Alter bzw. Einstiegsalter des Cannabiskonsums vollzogen, werden in jüngerer Zeit diese Parameter vermehrt erhoben. So bekommen die bisherigen Befunde, dass der frühe Beginn des Konsums, die Dauer, die Intensität, die Dosisstärke und die Häufigkeit des Konsums eine wesentliche Rolle für Auffälligkeiten sein dürften, zunehmendes Gewicht. Es gibt vermehrt Hinweise auf verminderte Gedächtnisleistung bei frühem Einstiegsalter und starkem Konsum. Auch nach Abstinenz zeigten besonders das verbale und visuelle Gedächtnis, das verzögerte Abrufen von Gedächtnisinhalten [57] und das räumliche Arbeitsgedächtnis [47] Auffälligkeiten. Konträr zu diesen Ergebnissen zeigt sich jedoch in einer Meta-Analyse von Schöller, dass die negativen Effekte auf das Gedächtnis umso geringer waren, je jünger die Cannabiskonsumenten im Vergleich zu den Kontrollprobanden waren [58].

Weiters konnte der frühe Konsumbeginn für die Einschränkung der Aufmerksamkeitsprozesse und für die Exekutivfunktionen im Sinne einer Störung der kognitiven Inhibition und der Inhibitionskontrolle und eben auch bei der Entscheidungsfindung im Sinne langfristiger Veränderungen nach längerer Abstinenz untermauert werden [47, 59].

Fundierte Aussagen zu Effekten nach Abstinenz bei frühem Beginn und chronischem Cannabiskonsum können derzeit nur mit Zurückhaltung getroffen werden, wobei jedoch die Tendenz der wissenschaftlichen Befunde in Richtung einer Schädigung wesentlicher Gehirnfunktionen bei Jugendlichen mit chronisch überdauerndem Konsum immer mehr Evidenz erhalten.

Die gewichtigsten Folgen stellen jedoch die Auswirkungen von frühem und intensivem Konsum im Sinne eines hohen Risikos auf andauernde Verringerung des IQ’s dar [51]. Auch bei funktionellen Hirnveränderungen unter kognitiver Beanspruchung zeigen sich trotz deutlicher Heterogenität der untersuchten Bereiche altersspezifischen Effekte, die auch nach längerer Abstinenz zu beobachten waren [59].

## Cannabis und Schwangerschaft

Tendenziell zeigt sich auch in der Schwangerschaft eine Zunahme des Cannabis-Konsums. In Europa stellt Cannabis die am häufigsten konsumierte, illegalisierte Droge in der Schwangerschaft dar. Entsprechend den Zahlen des European Monitoring Centre for Drugs and Drug Addiction [17] konsumieren mindestens 1–16 % der schwangeren Frauen Cannabis.

Studien mit Nagern zeigen, dass das eCB während der Embryonalentwicklung modulierend in wesentliche Reifungsprozesse des Gehirns eingreift, wie dem Überleben und der Proliferation neuronaler Stammzellen, der Entscheidung zur Zelldifferenzierung, der Migration, der Synaptogenese und dem axonalen Wachstum [60]. Auch wenn diese Untersuchungen vermutlich nur bedingt auf den Menschen übertragbar sind, geben sie doch Hinweise auf die Wichtigkeit des eCB für die neuronale Ausreifung während der Schwangerschaft [61].

Ein Problem vieler Studien zum Cannabiskonsum in der Schwangerschaft ist der überwiegende Zusatzkonsum von anderen Substanzen, hier in der Regel Nikotin, oder auch häufig Alkohol und andere Drogen. Die Befunde sind daher sehr heterogen, schwer vergleichbar und oft widersprüchlich. Cannabis ist kein klassisches Teratogen, jedoch wurden in einem Review [62] Zusammenhänge mit Missbildungen, wie Defekte des Gehirns, des kardiovaskulären oder des gastrointestinalen Systems gefunden. Dies konnte jedoch in anderen Untersuchungen nicht bestätigt werden. In neueren Untersuchungen [63] zeigten sich für Cannabiskonsumentinnen höhere Risiken für Frühgeburtlichkeit, vorzeitige Plazentalösung, ein „small for date“ und schlechtere Vitalparameter beim Kind peri- und postnatal. Außerdem fand sich ein signifikant erhöhtes Risiko für eine Anämie bei der Mutter. Außerdem wurden in diesem Review [64] Zusammenhänge zwischen Cannabiskonsum und erniedrigtem Geburtsgewicht und dem Risiko für intensivmedizinischer Behandlung erhoben.

In der „Ottawa prenatal prospective study“ [65] wurden rein nikotinkonsumierende Mütter mit solchen, die zusätzlich Cannabis konsumierten verglichen. Bei Neugeborenen von Müttern, die in der Schwangerschaft Cannabis konsumiert hatten, fand man veränderte Antworten auf visuelle Stimuli und ein verändertes Schreiverhalten. Dies könnte ein Hinweis auf intrauterine Einflüsse von Cannabiskonsum auf die neurologische Entwicklung sein. Hinsichtlich eines Einflusses auf die Intelligenz konnte bei Cannabis-exponierten Kindern im Gegensatz zu Cannabis-naiven Kindern keine Unterschiede gefunden werden, sehr wohl aber fand man Beeinträchtigungen im Bereich der Exekutivfunktionen, wie der Daueraufmerksamkeit und dem visuellen Gedächtnis [66].

Jugendliche, deren Mütter in der Schwangerschaft Cannabis konsumierten, zeigten eine höhere Rate an Delinquenz, Depressionen, Aufmerksamkeitsproblemen und Angststörungen. Die pränatale Cannabisexposition ist auch verbunden mit einer höheren Wahrscheinlichkeit, selbst in der Adoleszenz Cannabis zu konsumieren [67].

THC tritt bei Stillenden auch in die Muttermilch über und kann dort auch in hohen Dosen akkumulieren und die neurologische Entwicklung ungünstig beeinflussen [68]. Derzeit existieren noch wenige Daten über den Einfluss von Cannabis während der Schwangerschaft und des Stillens, wobei es jedoch Hinweise gibt, dass Fütterungsschwierigkeiten, Lethargie und verzögerte kognitive und motorische Entwicklungen beim Kind auftreten können [69]. Die meisten Studien sind auch deshalb nicht konklusiv, da die meisten auf „self-reports“ basieren. Ebenso scheinen die Einflüsse von Störvariablen wie etwa Nikotin- und Alkoholkonsum oder sozioökonomischer Status bedeutend zu sein, die oftmals keine adäquate Berücksichtigung fanden.

Zusammenfassend sei hier eine Stellungnahme des „American College of Obstetricians and Gynecologists“ [70] zitiert, das angesichts der zunehmenden Liberalisierung des Zugangs zu THC-hältigen Cannabisprodukten eindrücklich vor dem Konsum während Schwangerschaft und Stillzeit warnt. In dieser Zeit sollte auf den Konsum gänzlich verzichtet werden bzw. der Konsum so weit wie möglich reduziert werden.

## Cannabis und assoziierte psychische Störungen, Auswirkungen und Komplikationen

Ein Zusammenhang von psychischen Störungsbildern und Cannabis-Konsum im Jugendalter ist durch zahlreiche Studien belegt. In epidemiologischen Studien zeigen jugendliche Cannabiskonsumenten ein deutlich höheres Risiko für die Entwicklung eines missbräuchlichen oder abhängigen Konsums anderer Substanzen als erwachsene Konsumenten [71]. Dies zeigt sich auch in Forschungsergebnissen, die darstellen, dass Cannabis-Konsum bei Beginn im Jugendalter nach einer Latenzperiode von zwei Jahren etwa zwei bis vier Mal häufiger zu einer Cannabis-Abhängigkeit führt als bei Beginn im Erwachsenenalter [72]. Dies begünstigt wiederum das Risiko, andere illegale Substanzen zu konsumieren [73]. Erklärungsmodelle verweisen hierbei unter anderem auf veränderte Dopamin-Reaktivität im Belohnungssystem des Gehirns [74].

Auch psychische Störungsbilder abseits von Abhängigkeitserkrankungen sind mit frühem und intensivem Cannabiskonsum assoziiert, beispielsweise Depression und Angststörungen [75]. Neben dem allgemein erhöhten Risiko, paranoide und psychotische Gedanken zu entwickeln, zeigt sich bei Erkrankungen aus dem schizophrenen Formenkreis für Cannabis-Konsumenten eine signifikant schlechtere Prognose, insbesondere bei Personen mit erhöhter genetischer Vulnerabilität [76]. Beeinflussende und korrelierende Faktoren sind hierbei Dosis, Frequenz des Konsums sowie das Einstiegsalter [77]. Auch ein hoher THC-Gehalt scheint laut aktueller Studienlage mit einem erhöhten Psychoserisiko assoziiert zu sein [78]. Statistisch schwache Zusammenhänge finden sich zwischen Cannabiskonsum im Jugendalter und Suizidalität [79].

Abseits von klar umrissenen Störungsbildern sind bei Cannabiskonsum im Jugendalter Kurz- und Langzeitfolgen zu beobachten, welche sich schädlich auf die psychische und physische Gesundheit der Konsumenten auswirken können. Neben der naheliegenden Beeinträchtigung des Urteilsvermögens während der akuten Cannabis-Intoxikation, welche zu einer erhöhten Bereitschaft für risikoreiches Verhalten führt, beeinträchtigt Cannabis-Konsum bei Jugendlichen das Kurzzeitgedächtnis, die Feinmotorik und Koordination und stellt somit immer ein deutlich höheres Risiko für Autounfälle, Sachschäden oder Unfälle mit Todesfolgen, vor allem bei jugendlichen, unerfahrenen Führerscheinbesitzern, dar [80].

Bei hohen Dosierungen kann auch der Einmalkonsum zu akuter Paranoia, angstgefärbtem Erleben, Angst, Panik und Derealisations- sowie Depersonalisationsphänomenen führen. Es können sich auch kurze psychosewertige Symptome, insbesondere visuelle und auditive Halluzinationen einstellen [81, 82].

**Synthetische Cannabinoide** sind aufgrund der höheren Affinität am CB-1‑R und der vermutlich fehlenden partiell-antagonistischen CBD-Wirkung deutlich stärker wirksam als biologisches („natural grown“) Cannabis und können unter Umständen unkalkulierbare Symptome erzeugen, wie z. B. schwere Erregung, Hypertension, epileptische Anfälle, Myoklonien, Rhabdomyolyse, Erbrechen, Hypokaliämie. Diesbezüglich sind auch schon Todesfälle dokumentiert [83].

**Zu den Langzeiteffekten** von hochfrequentem Cannabiskonsum gibt es Studien, die auf eine potenzielle Neurotoxizität hinweisen. Nach der Studie von Kessler und Cangas zeigen sich Cannabisstörungen häufig in Koexistenz mit anderen psychischen Störungen [84].

Langzeitkonsum geht oft mit depressiven Symptomen, Suizidalität, bipolaren Störungen, Angsterkrankungen und dem Konsum anderer illegaler Drogen einher. Wie hoch der Anteil jener ist, bei denen Cannabis hier als Eigentherapie gegen Symptome einer zugrundeliegenden Erkrankung eingesetzt wird, lässt sich aufgrund der verfügbaren Studien nicht genau beziffern.

9 % aller Menschen, die Cannabis konsumiert haben, entwickeln eine cannabisbezogene Störung. Wenn der Konsum schon in der Adoleszenz begonnen hat, sind 17 % mit erhöhtem Risiko belastet. 25 % bis 50 % entwickeln eine Abhängigkeit, wenn Cannabis täglich konsumiert wird [81].

Ob der Zusammenhang mit niedrigerem Intelligenzniveau lediglich ein Maskieren von kognitiven Exekutivfunktionen bei hochfrequentem, über einen längeren Zeitraum anhaltenden Konsum abbildet und somit als reversible Auswirkung zu interpretieren ist, die bei Abstinenz ins Erwachsenenalter abnimmt, oder ob die Defizite hinsichtlich Lernens und Gedächtnis auch nach Drogenexposition persistieren, ist derzeit umstritten [85]. Es mehren sich aber die Hinweise, dass Beeinträchtigungen bei Beginn des Konsums im Jugendalter auch nach dem Beenden des Konsums noch bestehen bleiben [86]. In jedem Fall ist Cannabiskonsum im Jugendalter vor allem bei frühem Beginn mit deutlich schlechteren schulischen Leistungen, einem erhöhten Risiko für Schulabbruch, schlechteren Noten und weniger Abschlüssen, einer geringeren Rate an Studenten und weiteren negativen psychosozialen Folgen assoziiert [87]. Weitere Auswirkungen in später Adoleszenz und frühem Erwachsenenalter sind ein geringeres Einkommen, eine vermehrte Arbeitslosigkeit, Angewiesensein auf soziale Hilfen, geringere Zufriedenheit in Beziehungen und im Leben. Außerdem zeigen Konsumenten mit schweren psychischen Störungen eine erhöhte Delinquenz im Sinne der Ausübung von körperlicher Gewalt [88].

Zusammenfassend lässt sich festhalten, dass das Risiko von schädlichen Folgen und Auswirkungen des Cannabiskonsums klar mit früh einsetzendem, hochfrequentem und hochdosiertem Konsum im Jugendalter assoziiert ist. Dabei darf jedoch nicht außer Acht gelassen werden, dass die derzeitige Studienlage keine direkte Kausalität von frühem Cannabiskonsum auf die erwähnten Gesundheitsschäden beweist, sich die Hinweise darauf aber mit vermehrter Forschung verdichten. Somit können die beschriebenen Zusammenhänge innerhalb eines multifaktoriellen Modells gesehen werden, in dem neben dem Cannabiskonsum genetische Risikofaktoren sowie Umweltfaktoren beachtet werden müssen. Eine weitere Schlüsselrolle bei der Entstehung beschriebener Korrelationen könnte der Beeinflussung des körpereigenen eCB durch jugendlichen Cannabiskonsum zukommen, welche in der vulnerablen Schlüsselphase der adoleszenten Gehirnentwicklung besonders schädliche Auswirkungen hervorrufen kann.

## Medizinische Anwendung von Cannabinoiden im Kindes- und Jugendalter

In der umfassenden und empfehlenswerten Publikation „The Health Effects of Cannabis and Cannabinoids“ [89] wurde eine Bewertung der aktuellen Daten nach umfassender Auswertung qualitativ hochwertiger aktueller Literatur vorgenommen. Während der medizinische Einsatz von Cannabinoiden im Erwachsenenbereich in vielen Bereichen schon gut untersucht ist, ist die diesbezügliche Datenlage für Minderjährige noch rudimentär. Gerade in Hinblick auf diese Gruppe erwies sich ein Gutteil der kasuistisch kolportierten Wirkungen als anekdotisch und konnte bis dato nicht durch valide Studien bestätigt werden. Die bisherigen Erkenntnisse aus Untersuchungen im Erwachsenenbereich, welche im Folgenden kurz angeführt werden, können nur äußerst vorsichtig und unter Beachtung der speziellen Risiken in Hinblick auf die noch nicht abgeschlossene Hirnentwicklung auf Minderjährige umgelegt werden. Studien aus den Bereichen Onkologie, Ophthalmologie und die Neurologie mit den Schwerpunkten der neurodegenerativen Erkrankungen werden, da nicht der Zielgruppe der Publikation entsprechend, im Folgenden nicht dargestellt.

Eine prospektive Studie zur **Anorexia nervosa** [90] beschreibt unter Therapie mit Dronabinol 5 mg/d gegen Placebo eine Gewichtszunahme in der Verum-Gruppe, allerdings wird keine statistische Signifikanz erreicht. Es existieren zu diesem Thema keine validen Meta-Analysen.

Es existieren nur wenige valide Studien zur **Therapie der Angststörungen**. Eine Studie [91] untersucht die Kurzzeitwirkung von Cannabidiol (600 mg 1 ×) bei Patienten mit Sozialphobie und findet signifikant positive Ergebnisse. Weitere Studien von geringerer Qualität, welche eigentlich Patienten mit chronischem Schmerz untersuchten, fanden eine kurzfristige Verbesserung zusätzlich erhobener Angstsymptome unter Therapie mit Dronabinol, Nabilone oder Nabiximol. Im Gegensatz dazu fanden Beobachtungsstudien, auch mit jugendlichen Patienten [92–94], eine verstärkte Angstsymptomatik bis hin zur Entwicklung einer Sozialphobie vor allem bei höheren Dosen [95].

Es gibt derzeit keine valide Studie zur Wirkung von Cannabis bei **Depression** [89]. In Studien an Patienten mit chronischer Schmerzsymptomatik oder Multipler Sklerose unter Therapie mit Cannabis, in welchen auch depressive Symptome erhoben wurden, zeigten sich geringe positive Effekte, allerdings kam es in manchen Fällen auch zu einer Verschlechterung der depressiven Symptomatik.

Es gibt Studien zu Patienten mit Schlaf-Apnoe-Syndrom, Fibromyalgie, chronischem Schmerz und Multipler Sklerose [91]. Hier führte die Einnahme von Dronabinol und Nabiximol zu einer Besserung der **Schlafstörungen**. Auch hier gibt es noch keine hinreichend validen Daten, um auf eine Evidenz schließen zu können.

Ein Review-Artikel zur Wirkung von Cannabis bei **posttraumatischer Belastungsstörung** [96] kommt zu keinem Ergebnis hinsichtlich Besserung oder Verschlechterung unter Cannabis. Wenige Einzelstudien und „case series“ [97–99] mit geringen Fallzahlen, zeigten eine gewisse Wirkung von Cannabis und Cannabinoiden (Nabilone) hinsichtlich der Besserung der Symptomatik.

Zwei Übersichtsarbeiten zur **Therapie bei Schizophrenie** zeigen keine Besserung unter Therapie mit Cannabidiol, weder als Einzeltherapie, noch als add-on zur antipsychotischen Therapie (Amisulprid). Gleichzeitig wird aber auch auf das Risiko einer Aggravierung der Erkrankung unter Cannabinoiden verwiesen [91].

Ebenfalls existieren keine validen Daten zur Wirksamkeit und Sicherheit von Cannabinoiden bei **Autismus**. Präklinische Studien beschreiben Cannabinoide jedoch als vielversprechende Kandidaten in der Behandlung der Autismusspektrumsstörung [100, 101].

Lediglich eine kleine Placebo-kontrollierte Studie [102] mit 30 Patienten, beschäftigt sich mit der Behandlung von **adultem ADHS** mit Cannabis. Unter Therapie mit Nabiximol-Spray konnte in dieser Untersuchung eine signifikante Verbesserung der Hyperaktivität und Impulsivität erreicht werden, jedoch keine Verbesserung der Aufmerksamkeit.

In der **Therapie von Suchterkrankungen** wurde untersucht, inwiefern mit Hilfe von Cannabinoiden eine Cannabisabhängigkeit im Sinne einer Substitution therapiert werden kann. Eine Studie [103] zeigt gegenüber Placebo unter „Substitution“ mit Dronabinol eine geringere drop-out Rate, geringere Entzugserscheinungen aber keine signifikante Besserung hinsichtlich einer generellen längerfristigen Abstinenz. Ähnliche Ergebnisse wurden unter „Substitution“ mit Nabiximol [104] gefunden. Beide Studien wurden an Erwachsenen durchgeführt. Es gibt keine sichere Evidenz zur Wirksamkeit von Cannabis bei Cannabis-abhängigen Jugendlichen.

Zwei systematische Reviews [91, 105] beschreiben einen signifikant positiven Effekt auf das **Tourette-Syndrom** unter Therapie mit THC-Kapseln. Der Wirkmechanismus ist unklar, eventuell besteht ein Zusammenhang mit der angstlösenden Wirkung von THC. Es gibt also limitierte Evidenz für die Wirksamkeit von Cannabinoiden bei Tourette-Syndrom.

Es gibt zahlreiche evidenzbasierte Studien und Metaanalysen, die die Wirkung von Cannabis bei **chronischer Schmerzsymptomatik** belegen [91, 106, 107]. Die Mehrzahl dieser Studien zeigt Wirkung gegen neuropathischen Schmerz, eine geringere Zahl Wirkung gegen Schmerzen im Rahmen von Krebserkrankungen, Multipler Sklerose und rheumatoider Arthritis.

Zwei Meta-Analysen [105, 108] bei Erwachsenen mit verschiedenen **Epilepsie-Formen** beschreiben eine generell unbefriedigende Studienlage ohne eindeutige Ergebnisse. Case Reports an Patienten mit therapieresistenter Epilepsie [109–111] beschreiben eine deutliche Reduktion von epileptischen Anfällen unter Cannabinoiden. In der Behandlung der **juvenilen Epilepsieformen** des Dravet und Lennox-Gastaut Syndromes ist CBD eine mittlerweile gut untersuchte Substanz. Mehrere randomisierte, kontrollierte Studien konnten hier eine Wirksamkeit nachweisen, die derer klassischer Antiepileptika vergleichbar war [112]. CBD führte mitunter zu einer deutlicheren Reduktion von Anfällen als klassische Antikonvulsiva, zeigte allerdings auch eine höhere Rate von Nebenwirkungen wie Somnolenz, Appetitverminderung und Durchfall [113, 114].

**In Österreich erhältliche medizinische Cannabispräparate sind **magistraliter verordnete Dronabinol-Zubereitungen sowie Sativex®, Nabilone®, Cesamet®, Canemes® und Marinol®.

Dronabinol, welches in österreichischen Apotheken erhältlich ist, wird vorwiegend aus dem von der Agentur für Gesundheit und Ernährungssicherheit (AGES) produziertem Cannabis hergestellt. Das Cannabinoid THC wird von der Deutschen Arzneimittelfirma Bionorica Ethics aus diesen Pflanzen extrahiert. THC kann auch von einem zweiten deutschen Arzneimittelhersteller (THC Pharm) geliefert und von Apotheken zubereitet werden. Die Dronabinol Reinsubstanz wird in den Apotheken für Patienten in der Regel mit Sesamöl aufbereitet um es in Tropfenform gut dosieren zu können. Alkoholische Dronabinol-Lösungen stellen eine weitere Verarbeitungsmöglichkeit der Apotheken dar. Diese können nicht nur oral eingenommen, sondern auch mittels eines Verdampfers (Vaporizer) inhaliert werden.

Die erheblichen Kosten werden derzeit von den Krankenkassen äußerst zurückhaltend übernommen und ausschließlich über chefärztliche Genehmigung. In den USA und der EU ist weiters Epidiolex® für die adjuvante Behandlung von Krampfanfällen, im Zusammenhang mit Lennox-Gastaut-Syndrom oder Dravet-Syndrom zugelassen.

### Fazit

Zumindest im Erwachsenenalter besteht Evidenz für die Wirksamkeit von Cannabispräparaten bei chronischer Schmerzsymptomatik, Übelkeit und Erbrechen unter Chemotherapie und Spastizität bei Multipler Sklerose. Für alle anderen Störungsbilder gibt es ungenügende oder keine Evidenz. Als vielversprechend wird eine mögliche Wirksamkeit von Cannabis bei PTSD und Epilepsie angenommen.

Für Kinder und Jugendliche besteht in keiner Indikation ausreichende Evidenz. Der Einsatz von Cannabisprodukten bei Minderjährigen sollte nur ausnahmsweise und im Einzelfall nach genauer Abwägung des Nutzens gegen die Risiken (wie etwa Beeinflussung der Hirnentwicklung, erhöhtes Risiko für Psychosen, Suchtentwicklung) überlegt werden.

Insgesamt bedarf es aber weiterer Studien, insbesondere auch mit klarer Abgrenzung der Indikation, Wirkung und Nebenwirkungen bei Kindern und Jugendlichen.

## Therapie des Cannabis-Konsums

### Therapieprinzipien

Im Jugendalter wird der Behandlungswunsch viel häufiger durch Angehörige an die Behandler herangetragen, als durch die Jugendlichen selbst. Zu Behandlungsbeginn finden sich naturgemäß häufig massive familiäre Konflikte, wobei sich im weiteren Behandlungsverlauf doch oft ein hoher Wunsch nach elterlicher Unterstützung entwickelt. Der Beginn einer Behandlung wird im Kindes- und Jugendalter oftmals durch das Verleugnen und Verharmlosen des Konsums durch den Jugendlichen, aber auch durch das familiäre und engere Bezugssystem, wie Freunde, Lehrer, Ausbildner usw. verzögert. Ebenso wirkt sich der ungebrochene Kontakt zu konsumierenden Gleichaltrigen, mangelnde Einsicht und ein nicht vorhandener Konsumverzicht negativ auf den weiteren Verlauf aus [115].

Zusätzlich sind die über die reine Suchtbehandlung hinausgehenden psychosozialen Behandlungsnotwendigkeiten stärker im Vordergrund als im Erwachsenenbereich, insbesondere die schulische und berufliche Re-Integration sowie die entsprechende pädagogische Führung.

Nach Thomasius [116] gilt es, die vier unterschiedlichen Ebenen einer substanzbezogenen Störung zu behandeln:

Behandlung der körperlichen Auswirkung des Substanzmissbrauchs, wie Entzugssymptomatik, aber auch bei zusätzlichem Missbrauch anderer Substanzen, sowie körperlich begleitend Folgeerkrankungen des Substanzmissbrauchs.

Behandlung der psychischen Funktionsstörung, wie Problemlösungsstörung, Wahrnehmungsstörung, Ausdrucksstörung, emotionale und Motivationsstörung, sowie Störung der Psychomotorik.

Behandlung der Entwicklungsstörungen, wie fehlende Lebensperspektive, abgebrochene Schul- und Berufsausbildung.

Diagnostik und Behandlung komorbider psychischer Störungen.

Prochaska und Di Clemente konzeptualisierten einen zunehmenden Veränderungsprozess in fünf Phasen, den der Klient im Zuge seines Kontaktes mit Behandlern durchläuft [117]. Gerade bei jugendlichen Konsumenten besteht oft eine längere Phase der Präkontemplation mit fehlender Veränderungsbereitschaft, gefolgt von der Kontemplation mit zunehmender Einsichtsbereitschaft und Reflektion der Reaktionen aus der Umwelt. Anschließend stellt sich eine ersthafte Veränderungsbereitschaft in der 3. Phase ein, in welcher der Konsument ernsthafte Behandlung erwägt. Die Phase 4 ist geprägt von erreichten Veränderungen, die Behandlungsziele werden teilweise erreicht. In der Phase 5 geht es um die Aufrechterhaltung der Abstinenz mit Rückfällen, die eine längere Zeit von erneutem Substanzmissbrauch bedeuten. Somit sind auch Rückfälle, insbesondere im Jugendalter, als Teil eines Veränderungsprozesses zu verstehen, Abstinenzphasen werden bei entsprechender Motivationshaltung zunehmend länger.

Ein weiteres wichtiges Konzept in der Behandlung jugendlicher Konsumenten ist das Behandlungsmodell der „Motivierenden Gesprächsführung“ oder „Motivational Interviewing“ (MI), entwickelt von Miller & Rollnik [118]. Eines der wichtigsten Ziele des MI ist die Herausarbeitung der Diskrepanz zwischen dem Weiterführen des Problemverhaltens und den mittel- bis langfristigen persönlichen Zielen des Jugendlichen.

Die Behandlung der Abhängigkeitserkrankung teilt sich in eine Akut- und Postakutbehandlung von bis zu mehreren Wochen zur Entgiftung und in eine Entwöhnungsbehandlung. Diese ist ambulant oder, vor allem nach mehreren frustranen Behandlungsversuchen, insbesondere auch bei schwerwiegenden Komorbiditäten, stationär an einer Abteilung für Kinder- und Jugendpsychiatrie durchzuführen. Angeschlossen werden entsprechende Nachsorgemaßnahmen der Festigung des Behandlungserfolges, der Rückfallprophylaxe und entsprechender Re-Integrationsmaßnahmen. Hier finden Schul- und Berufsausbildung, psychotherapeutische Unterstützung, sowie regelmäßige, oftmals modular aufgebaute Nachsorgeprogramme ihre Einsatzgebiete.

Des Weiteren kommen Motivationsarbeit, Psychoedukation, Psychotherapie, körperbezogene Verfahren in Einzel- und Gruppensettings, Familientherapie und Rückfall-Präventionstraining zur Anwendung. Ebenso Bewegungs- und Körpertherapie, Musiktherapie, Ergotherapie und Kunsttherapie. Diese wesentlichen Behandlungsprinzipien haben sich über die letzten Jahre in der klinischen Erfahrung in der ambulanten und stationären Kinder- und Jugendpsychiatrie für eine Entwöhnungsbehandlung empfohlen. Die Therapie sollte intensiv und lang genug veranschlagt sein, um eine dauerhafte Beendigung des Substanzmissbrauchs zu erzielen und auch die Behandlung etwaiger Komorbiditäten miteinbeziehen [119]. Es bedarf der intensiven Einbeziehung der Herkunftsfamilie mit einer Stärkung der erzieherischen Kompetenz der Eltern. Intensität und Dauer der Behandlung sollten den individuellen Voraussetzungen des Patienten entsprechen und sollten alle dysfunktionalen Lebensbereiche umfassen. Der Besuch von Selbsthilfegruppen soll angeregt werden. Die Behandlung muss sozioökonomisch und kulturell entsprechend der Voraussetzungen des Patienten ausgerichtet sein. Eine spezielle Nachsorgebehandlung sollte rechtzeitig initiiert werden.

### Akuttherapie

Der Cannabisentzug geht in der Regel mit verschiedenen Symptomen aus dem Bereich von Craving, Schlafmangel, Albträumen und bizarren Träumen, innerer Unruhe, erhöhter Irritabilität und Reizbarkeit, Dysphorie, Aggression, Nervosität, Appetit- und Gewichtsverlust, Angst und Hyperalgesie und Hyperhidrose einher. Seltener sind Schüttelfrost, Depressivität, Magenschmerzen, Tremor und Schwitzen, sowie weitere vegetative Symptome (Bauchschmerzen, erhöhte Temperatur, Kälteschauer, Kopfschmerzen). Die Symptome sind in ihrer Intensität sehr variabel und hängen auch von der subjektiven Belastbarkeit ab. Sie wurden in der ersten Woche am intensivsten beschrieben und dauerten bis zu einem Monat an [120].

#### Die Behandlung von Cannabis-assoziierten Störungen

erfordert grundsätzlich immer eine umfassende kinder- und jugendpsychiatrische Diagnostik, insbesondere zur Erfassung von etwaigen Komorbiditäten. Ebenso sollte eine somatische Abklärung erfolgen. Oftmals reichen für diese Phase allgemeine sozialtherapeutische Maßnahmen. In einer geringen Anzahl von Fällen ist der symptomorientierte Einsatz von Medikamenten zur Unterstützung notwendig. Hier kommen schlaffördernde Antidepressiva, niedrigpotente Neuroleptika sowie Antihistaminika zur Behandlung der Schlafproblematik, der Reizbarkeit und der inneren Unruhe zum Einsatz. In besonders schweren Fällen können kurzfristig Benzodiazepine Linderung verschaffen. Hier ist jedoch auf das zusätzliche Abhängigkeitspotential zu achten, ein Einsatz von mehr als drei Wochen ist zu vermeiden. Kurzfristige „Cannabispsychosen“, die ähnlich einer akuten psychotischen Episode einer schizophrenen Störung verlaufen können, sind in entsprechenden unterstützenden Settings, eventuell mit dem Einsatz von Neuroleptika gut beherrschbar. Hier kann eine stationäre Aufnahme notwendig sein, um Abstinenz sichernde und beschützende Maßnahmen zu gewährleisten. Darüber hinaus bedarf es für den Patienten selbst, aber auch sein engeres Umfeld einer entsprechenden Psychoedukation und Förderung der Krankheitseinsicht. In wie weit im Jugendalter Cannabidiol (CBD) mittelfristig bei Craving-Symptomen einsetzbar ist bleibt vorerst entsprechenden Studien und klinischen Erfahrungen vorbehalten.

Die in der Literatur oftmals beschriebenen Konzentrations- und Aufmerksamkeitsstörungen nach chronisch hochfrequentem Langzeitkonsum bei Jugendlichen bedürfen einer sicheren Abstinenz. In der Regel bessern sich die Symptome, unterstützt durch eine Tagesstruktur mit individuell angepassten schulischen oder beruflichen Anforderungen. Zusätzlich kann noch eine Lerntherapie oder kognitiv fördernde Ergotherapie angewendet werden. Wir verweisen in diesem Zusammenhang auf die Ausführungen zu den kognitiven Auswirkungen (Kapitel „Cannabis und assoziierte psychische Störungen, Auswirkungen und Komplikationen“) von frühem Cannabiskonsum. Hier bedarf es wiederum abstinenzfördernder und psychosozial aktivierender Maßnahmen und Psychoedukation. Die Symptomatik bessert sich oftmals zum Teil über einen längeren Zeitraum, häufig bleibt aber eine gewisse Restsymptomatik bestehen.

#### Spezifische cannabisbezogene Behandlungsprogramme

[121] beinhalten folgende Komponenten: Rückmeldung der diagnostischen Ergebnisse, Vermittlung eines ätiologischen Models der Cannabisstörung, Ableitung einer Behandlungsrationale, Aufbau von Veränderungsmotivation, Förderung von Einsicht in die Problematik, systematische Selbstbeobachtung, Aufbau von Fertigkeiten zur Beendigung des Cannabiskonsums (Skills-Training), Behandlung von Entzugssymptomen, Umgang mit starkem Verlangen und Behandlung komorbider psychischer Störungen.

### Postakutbehandlung der Cannabistherapie

Hier werden in modular aufgebauten Behandlungsprogrammen, basierend auf der Kombination von Motivationsförderung, kognitiv behavioraler Therapie und Kontingenzmanagement die Möglichkeiten eines abstinenten Lebens und die daraus folgende Rückfallprophylaxe erarbeitet.

Ähnlich aufgebaute Programme wie „Quit the Shit“ (https://www.quit-the-shit.net/qts/fwd/public/programm.do) oder „Realize it“ (http://www.suchthilfe.ch/cannabisberatung-realize-it.html) sind niederschwellige online Therapieprogramme. Darüber hinaus wurden gruppentherapeutische Therapieprogramme entwickelt, wie etwa „CAN STOP“, welches u. a. im Jugendstrafvollzug zur Anwendung kommt. Zusätzlich wurden für die Zielgruppe der jugendlichen Erst- und Intensivkonsumenten mit massiven psychosozialen Problemen hochspezialisierte, pädagogische Programme entwickelt, wie z. B. die MST – Multisystemische Therapie [122], in denen auch Eltern und Umfeld intensiv eingebunden sind.

## Rechtlicher Rahmen, Rechtliche Situation in Österreich

Cannabis, als „psychoaktive Substanz“, wird in österreichischen Gesetzen zu den „Suchtgiften“ gezählt. Der Umgang mit diesen ist in Österreich durch die Bestimmungen des Suchtmittelgesetzes [123] geregelt. Die Einstufung von Cannabis als Suchtmittel stützt sich in Österreich auf das UNO Einheitsabkommen [124] über die Betäubungsmittel. Der Umgang mit THC-haltigem Cannabis ist in Österreich strafrechtlich verboten [125]. Ausnahmen sind Anwendungen aufgrund medizinisch indizierter ärztlicher Verordnung sowie für aufsichtsbehördlich genehmigte Forschungszwecke. Diese Ausnahme für die Produktion von medizinischem Cannabis besteht nur für die Agentur für Gesundheit und Ernährungssicherheit (AGES). Die AGES steht im Eigentum der Republik Österreich, vertreten durch den Bundesminister für Soziales, Gesundheit, Pflege und Konsumentenschutz. Seit einer Gesetzesnovelle im Jahr 2008 [123] ist es der AGES erlaubt, medizinisches Cannabis anzubauen und an befugte Abnehmer, in der Regel Unternehmen der Pharma-Branche, weiterzugeben. Hierzu wurde im Jahr 2008 der §6a geändert. Es besteht somit in Österreich ein Monopol des Staates zur Herstellung von medizinischem Cannabis.

Parallel zum Strafrechtsbestand gilt jedoch auch noch das Prinzip „Therapie statt Strafe“.

Im Konkreten gilt nach aktuellem Stand grob:

- „Straffreiheit“ (bei geringen Konsumdelikten in der Regel für Personen, die keiner gesundheitsbezogenen Maßnahme bedürfen).
- „Therapie statt Strafe“ (bei geringen Konsumdelikten und für Delikte im Sinne von Beschaffungskriminalität durch Suchtkranke für Personen, die einer gesundheitsbezogenen Maßnahme bedürfen).
- Suchtgiftdelikte, für die Straffreiheit oder „Therapie statt Strafe“ nicht in Frage kommen.

Besonders für das Jugendalter verweisen wir auf die Paragraphen 11, 12 und 13 des Suchtmittelgesetzes [123]. In diesem Zusammenhang halten wir folgende Ausführungen für relevant: Jugendliche, die Cannabis konsumieren, sollten einer notwendigen Beratung, Information im Sinne von „harm reduction“ und „safer use“ (Konsum- u. Risikokompetenz), Abklärung, Diagnostik und ggf. Behandlung und Therapie zugeführt werden.

Daraus sollte sich aus kinder- u. jugendpsychiatrischer Sicht die Notwendigkeit einer Entkriminalisierung konsumierender Minderjähriger ergeben.

Die Debatte der Legalisierung und Regulierung muss einer differenzierten und mehrdimensionalen bio-psycho-soziokulturellen Grundlage unter Einbeziehung verschiedenster Komorbiditäten entsprechen.

In diesem Zusammenhang ist auch die Behandlung mit medizinischen Cannabinoiden zu sehen, z. B. sollte eine Behandlung einer schweren Cannabisabhängigkeit bei Minderjährigen mit psychiatrischen Komorbiditäten als „individueller Heilversuch“ möglich sein, entsprechend der Opioidsubstitutionstherapie [126] bei opioidabhängigen Jugendlichen.

## Zusammenfassung

Cannabis Sativa ist eine der ältesten Kulturpflanzen der Welt. Bis heute wurden mehr als 100 verschiedene Cannabinoide, also psychoaktive chemische Bestandteile der Cannabispflanze, identifiziert. Zu den relevantesten zählen hierbei THC und CBD. Ein Nachweis über den Konsum von Cannabinoiden ist im Wesentlichen im Urin und Blut sinnvoll. Der menschliche Körper ist von Strukturen des eCB, des sogenannten „Endocannabinoid-Systems“ durchdrungen, über welches Cannabinoide ihre Wirkung entfalten können. Aus großen internationalen Studien geht hervor, dass Cannabis in Österreich und auch in den wesentlichen Industriestaaten die am häufigsten konsumierte illegalisierte Droge ist. Die Lebenszeitprävalenz des Cannabiskonsums liegt bei älteren Jugendlichen und jungen Erwachsenen in diesen Breiten bei annähernd 40 % und auch die Beschaffung wird als relativ einfach wahrgenommen. Die in den gängigen Diagnoseschemata definierten Störungsbilder reichen von „Konsum“ über „missbräuchlichen Konsum“ bis zu „Abhängigkeit“, die jedoch für jugendliche Konsumenten sehr unpräzise sind und eine große diagnostische Unschärfe beinhalten. Zu den Auswirkungen eines regelmäßigen Cannabiskonsums in der Phase der Entwicklung zählen neben neurophysiologischen und neuroanatomischen Veränderungen auch eine Veränderung von Konzentration und Verteilung der Neurotransmitter. Diese Veränderungen scheinen umso drastischer zu sein, je jünger die Konsumenten sind. Bei THC-Konsum in der Schwangerschaft zeigen sich bei den betroffenen Kindern unter Anderem veränderte Exekutivfunktionen und eine erhöhte Rate an Depressionen und Angststörungen. Konsumieren Jugendliche Cannabisprodukte, so haben sie im Vergleich zu Erwachsenen ein deutlich höheres Risiko im weiteren Verlauf eine Abhängigkeitserkrankung, sowohl von Cannabis als auch anderen psychotropen Substanzen, zu entwickeln. Auch die Raten an Angststörungen, Depressionen und Erkrankungen aus dem schizophrenen Formenkreis sind bei diesen Jugendlichen erhöht. Synthetische Cannabinoide stellen hierbei ein besonderes, weil unkalkulierbares Risiko dar.

Cannabisprodukte wurden und werden auch hinsichtlich ihrer medizinischen Anwendbarkeit untersucht. Während es hierbei im Erwachsenenbereich bereits einige Daten zum Einsatz im psychiatrischen Bereich, wie etwa bei Angst- oder Essstörungen, Depressionen, Schlafstörungen oder PTSD gibt, sind Cannabinoide im Kindes- und Jugendalter hauptsächlich im Bereich der Epilepsie im Einsatz.

Die Behandlung eines problematischen Cannabiskonsums wiederum fußt neben der Behandlung der körperlichen und psychischen Auswirkungen auch auf eine Therapie der Entwicklungsstörung sowie der psychosozialen Folgen und Komorbiditäten.

## Conclusio

Während gelegentlicher Freizeitkonsum von Cannabis bei Erwachsenen mit abgeschlossener Hirnreifung und ohne Risikoprofil für psychische Störungen relativ harmlos sein dürfte, ist die Datenlage bei Jugendlichen eine andere. Rezente Studien mit einem besonderen Fokus auf Jugendliche bringen zunehmend Gewissheit, dass wir hier eine neue Einschätzung vornehmen müssen. Der Einfluss von frühzeitigem Konsumbeginn und regelmäßigem Konsum sowie der zunehmend verfügbaren, hochpotenten Cannabis-Sorten auf das sich entwickelnde, juvenile Gehirn scheint weitaus weniger harmlos zu sein als bisher angenommen und kann zu expliziten und zum Teil irreversiblen neurokognitiven Hirnfunktionsstörungen führen.

Uns stehen bis dato keine Untersuchungsmethoden zur Verfügung, die Jugendliche mit einem hohen Risiko, eine derartige Störung zu entwickeln, identifizieren könnten. Dies gilt darüber hinaus auch für den Bereich früh einsetzender, schizophrener Störungen und sozialer Angststörungen, bei welchen der Cannabisgebrauch einen wichtigen Risikofaktor für die Entwicklung und Chronifizierung der Erkrankung darstellt.

Berücksichtigt man die aktuell zur Verfügung stehenden, seriösen, Daten ist also eine gesetzliche Freigabe des Cannabis-Konsums für Jugendliche aufgrund der zu erwartenden Schäden im Bereich der Gehirnentwicklung aus kinder- und jugendpsychiatrischer Sicht abzulehnen.

Zugleich gilt es aber, vernünftige gesetzliche Regelungen zu finden, um der Tatsache, dass über 30 % aller europäischen Jugendlichen gelegentlich Cannabis konsumieren, adäquat begegnen zu können. Wir sprechen uns hier auch klar dafür aus, Cannabiskonsumenten nicht zu kriminalisieren und gefährdeten und suchtkranken Konsumenten die benötigte Unterstützung zukommen zu lassen. Zusätzlich erachten wir die weitere Stärkung der Suchtprävention in Bezug auf Cannabis und das Jugendalter, insbesondere unter Einbindung der Kinder- und Jugendpsychiatrischen Expertise, für hochgradig relevant.

Zum medizinischen Einsatz von Cannabis ist festzuhalten, dass es für Kinder- und Jugendliche derzeit kaum Evidenz gibt, die einen breiten Einsatz von Cannabis und Cannabinoiden im psychiatrischen Bereich rechtfertigen würde. Aktuell laufen zahlreiche Studien, zum gegenwärtigen Zeitpunkt wäre es aber verfrüht, einen Einsatz im therapeutischen Bereich für diese Altersgruppe in einer spezifischen Indikation zu empfehlen.

Einzig in der Behandlung schwerer Cannabis-Abhängigkeitserkrankungen können Cannabis-Substitutionstherapien in Erwägung gezogen werden. Dies sollte jedoch nur suchtmedizinisch erfahrenen Kinder- und Jugendpsychiatern vorbehalten sein.

## Footnotes

1 Die verwendeten geschlechtsspezifischen Bezeichnungen beziehen sich natürlich auf alle Geschlechtsformen, auf eine durchgehende genderneutrale Formulierung wurde zugunsten einer besseren Lesbarkeit verzichtet.
